# Supplementary figures and images for: Localization and functions of native and eGFP-tagged capsid proteins in HIV-1 particles
Source: PLoS Pathog. 2022 Aug 11;18(8):e1010754. doi: 10.1371/journal.ppat.1010754 (PMC9426931; doi:10.1371/journal.ppat.1010754)

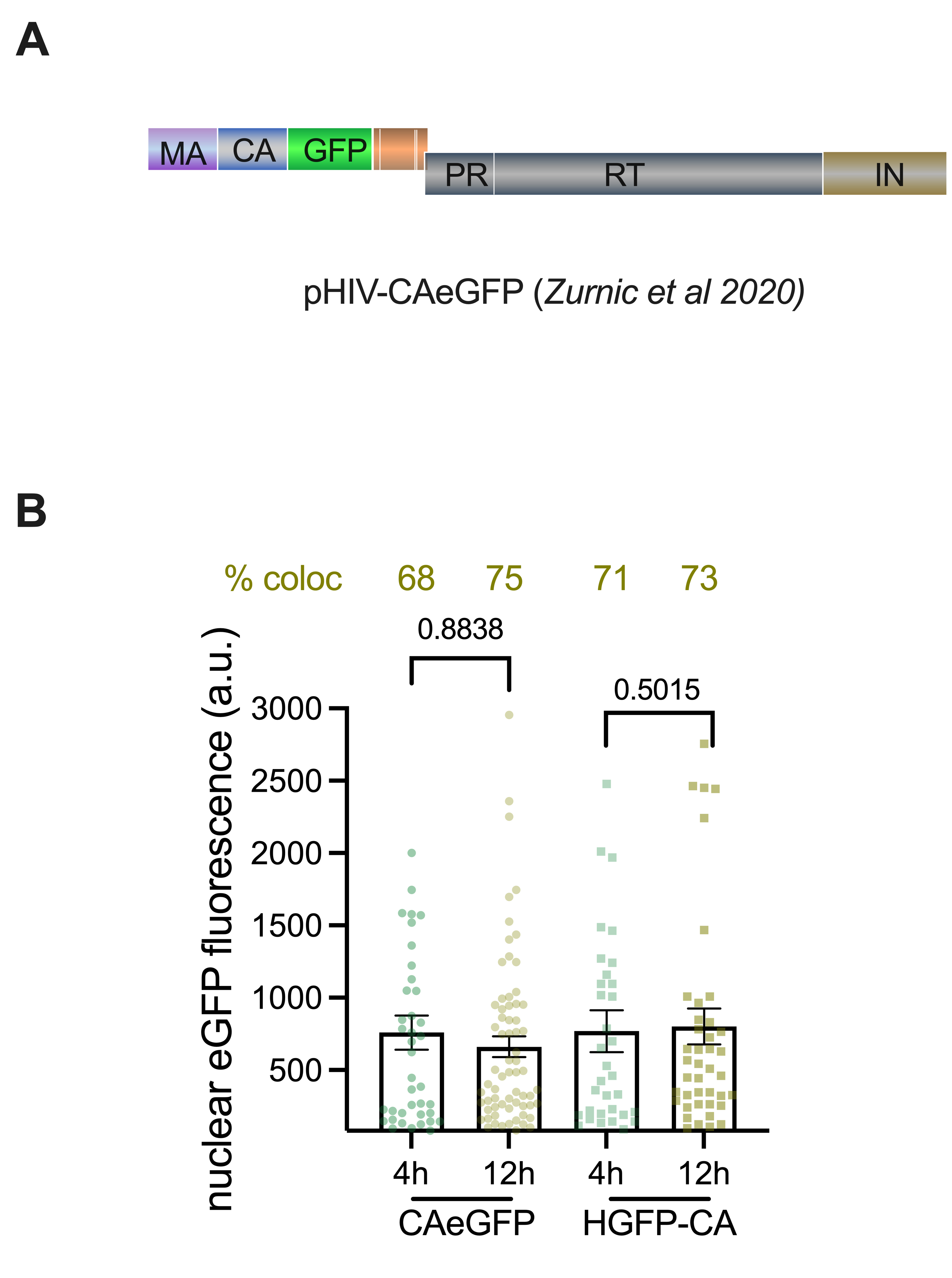

Supplement: S1 Fig — (A) Schematics of C-terminally tagged CAeGFP in the context of full-length Gag/Gag-Pol reported in (Zurnic et al, J. Virol 2020). (B) Fluorescence intensity analysis of eGFP-tagged CA signals in nuclear VRCs at 4 and 12 hpi was determined in TZM-bl cells, as reported in Fig 1G and 1H. (TIFF) [file ppat.1010754.s001.tiff]

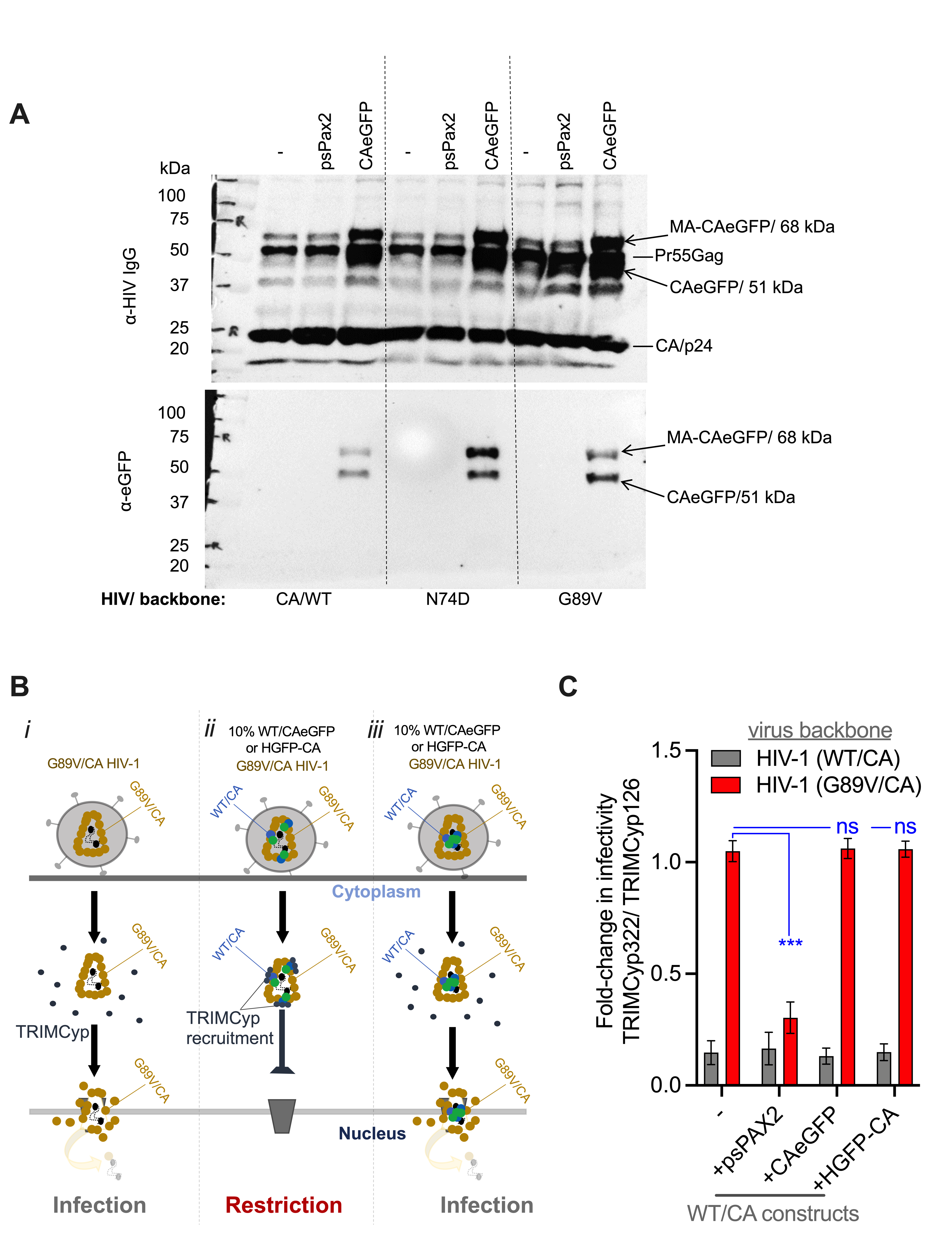

Supplement: S2 Fig — (A) SDS-PAGE of virus lysates (1ng of p24) of indicated HIV-1 CA WT and mutant viruses mixed with 10% of CAeGFP or unlabeled psPax2 CA probed with an anti-HIV-1 IgG (top) and anti-eGFP antibodies (bottom). Immunoblot shows efficient processing of MA-CAeGFP in virions (top) resulting in appearance of CAeGFP band (bottom). Putative bands corresponding to MA-CAeGFP, Gag/pr55 precursor, CAeGFP, and CA/p24 are marked. Note, in top HIV-IgG blot the processed CAeGFP (51 kDa) co-migrates with the prominent Pr55Gag band. (B) Strategy to test the localization of GFP-tagged CA in virions. HIV-1 containing G89V mutant CA are labeled with GFP-tagged WT/CA protein, and the ability of GFP-tagged CA to bind cytosolic TRIMCyp restriction factor constitutively expressed in target TZM-bl cells. The following scenarios are illustrated: (i) The G89V/CA mutant virus does not interact with TRIMCyp and naturally escapes restriction. (ii) If the virus-incorporated GFP-tagged WT/CA co-assembles on the capsid lattice, these proteins will interact with TRIMCyp and restrict G89V infectivity. (iii) If GFP-tagged CA is inaccessible to TRIMCyp, likely due to packaging into vRNP, G89V viruses will not be restricted. (C) Single-round infectivity data shows that GFP-tagged CA is inaccessible to TRIMCyp, while 10% unlabeled WT/CA in G89V viruses is recognized and restricted by TRIMCyp. The control HIV-1 infections with 100% unlabeled WT/CA or MT/CA were completely susceptible or escaped TRIMCyp restriction, respectively. Infectivity was normalized to control cells expressing the TRIMCyp126 mutant which does not interact with CA. The means and STDs from 4 experiments is shown. Statistics: in (C) student’s t-test was used. (TIFF) [file ppat.1010754.s002.tiff]

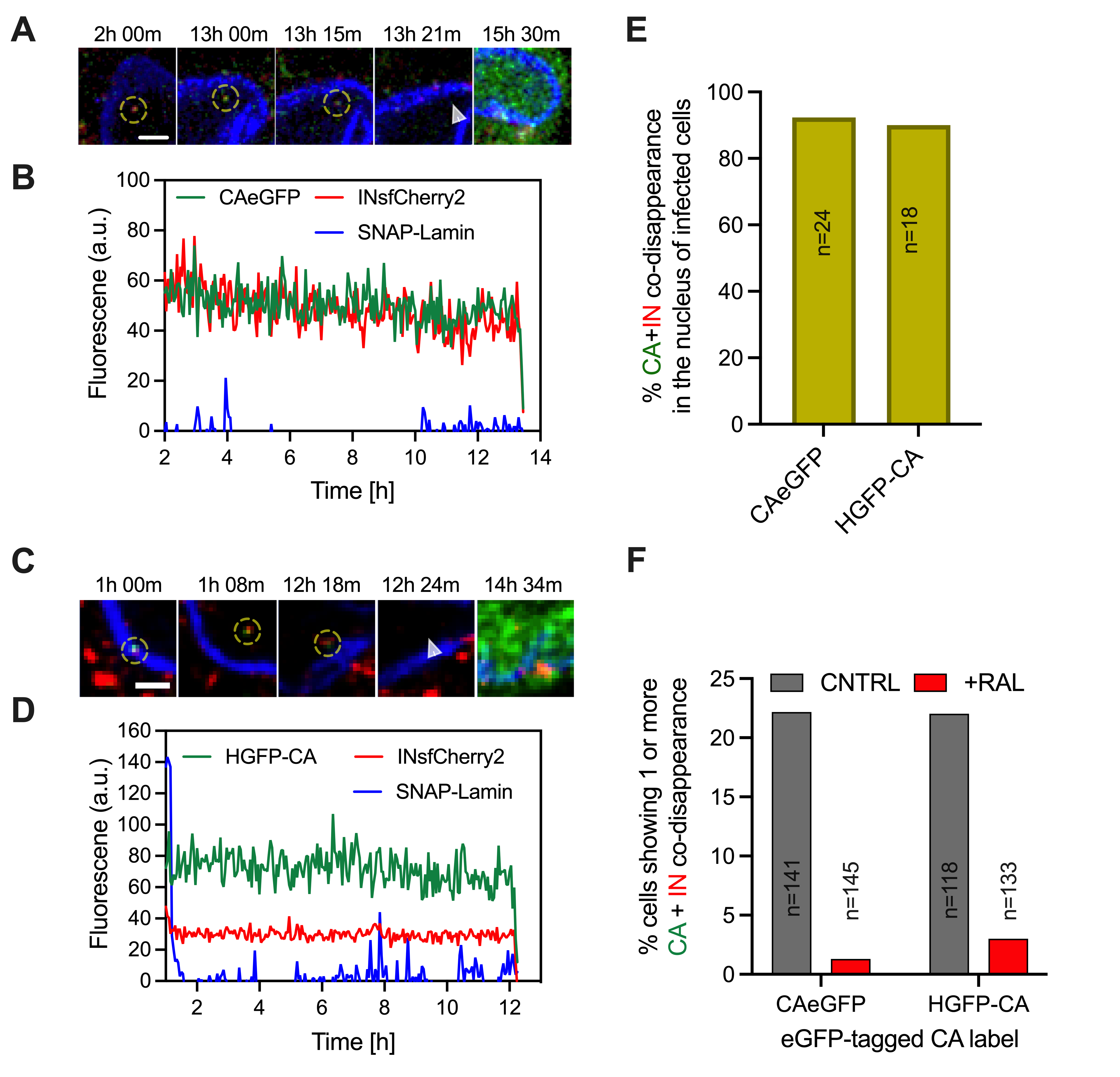

Supplement: S3 Fig — TZM-bl cells constitutively expressing SNAP-lamin nuclear membrane marker were infected (MOI 0.2) with HIV-pseudoviruses co-labeled with indicated GFP-tagged CA proteins and INsfCherry2 marker. Live-cell imaging was performed starting from 1 h and continued up to 22 hpi. Infected cells were identified by the expression of eGFP reporter and analyzed for nuclear INsfCherry2/GFP-tagged CA complexes, and these single viral complexes were tracked. (A, C) Images and (B, D) single virus fluorescence trace of examples shown in respective images. The images in (A, C) show HIV-1 complexes entering the nucleus (dashed yellow circles) trafficking for several hours and co-disappearing (arrowhead) followed by eGFP reporter expression. Fluorescence traces in (B, D) show that eGFP-tagged CA signal remains steadily associated with INsfCherry2 puncta and co-disappears at 13 h 21 min (A, B) and 12 h 24 min (C, D). Scale bar in A, C is 2 μm. Quantification of: (E) HIV-1 puncta that disappeared in the nucleus of infected cells and contained both CA- and IN- signals, and (F) the fraction of cells showing eGFP/sfCherry puncta disappearing in the nucleus by 22 h in control (CNTRL) non-treated or in the presence of integration inhibitor raltegravir (+RAL, 10 μM). Data is cumulative from 2 independent 22 h live-imaging experiments and (n) number of cells analyzed is shown. (TIFF) [file ppat.1010754.s003.tiff]

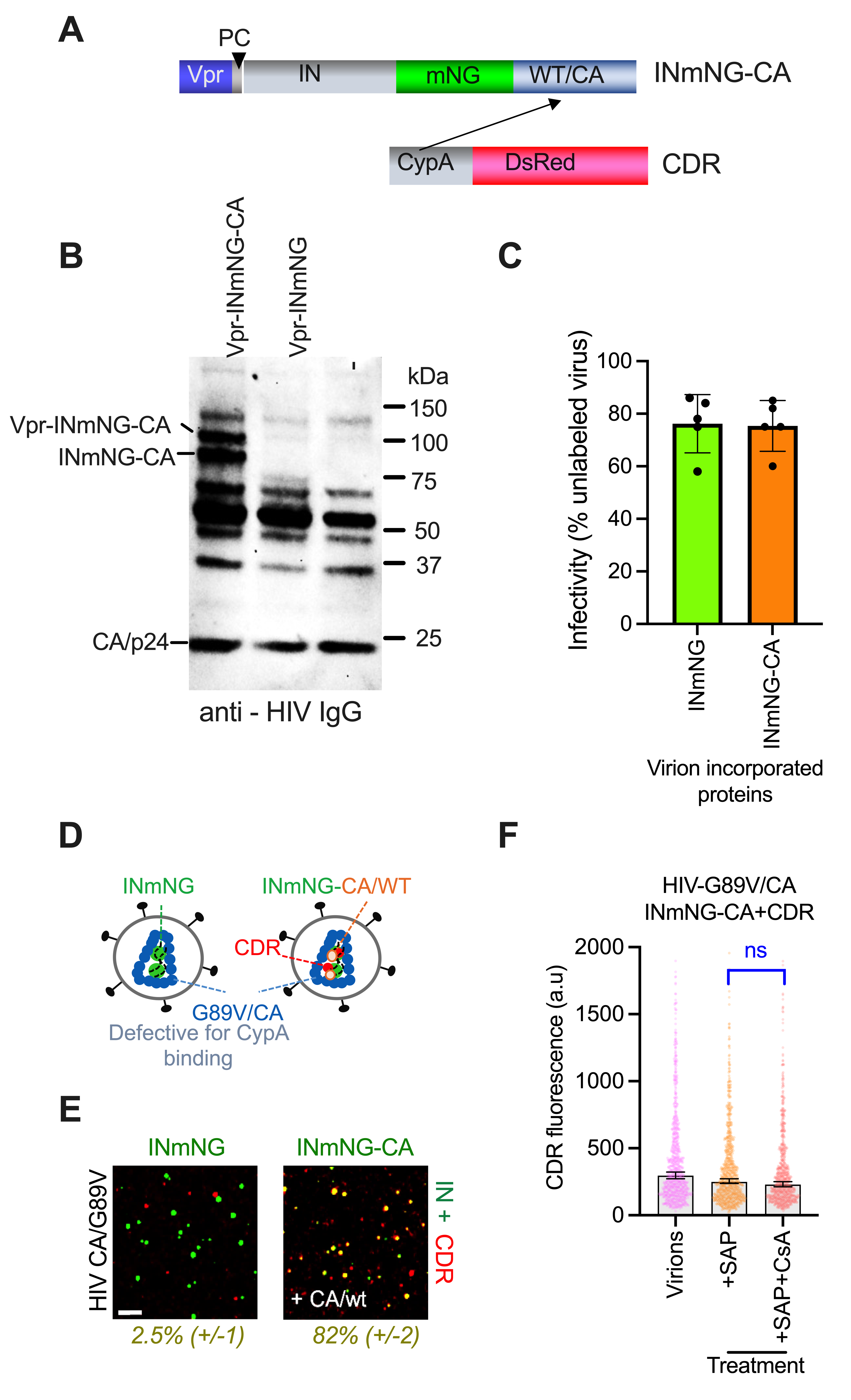

Supplement: S4 Fig — (A) Schematics of the chimeric INmNG-CA fusion construct, the protease cleavage site (PC: IRKVL/FLDGI) between Vpr- and IN is included to release INmNG-CA from Vpr after virion incorporation. (B) Immuno-blot analysis of unlabeled and INmNG or INmNG-CA labeled HIV-1 using anti-HIV-IgG antibodies. The migration of Vpr-INmNG-CA and processed INmNG-CA is marked. (C) Single round-infectivity of INmNG or INmNG-CA labelled HIV-1 with respect to unlabeled HIV-1 was measured using a luciferase assay in TZM-bl cells at 48 hpi (MOI 0.5). Dots represent independent triplicate experiments. (D) Schematic for the incorporation of CDR selectively with vRNPs in G89V CA mutant virus through co-incorporation with INmNG-WT CA fusion proteins. (E) Images showing lack- or efficient-labeling of G89V virus with CDR in the presence of INmNG control or INmNG-CA fusion protein, respectively. (F) Quantification of CDR signal in INmNG-WT CA labeled G89V virions or after SAP (+SAP) and CsA treatment shows insufficient release of CDR from cores. Data in (C and F) is shown as mean and SEM. Stats, Student t-test (C) and non-parametric Mann-Whitney Rank sum test (F). (TIFF) [file ppat.1010754.s004.tiff]

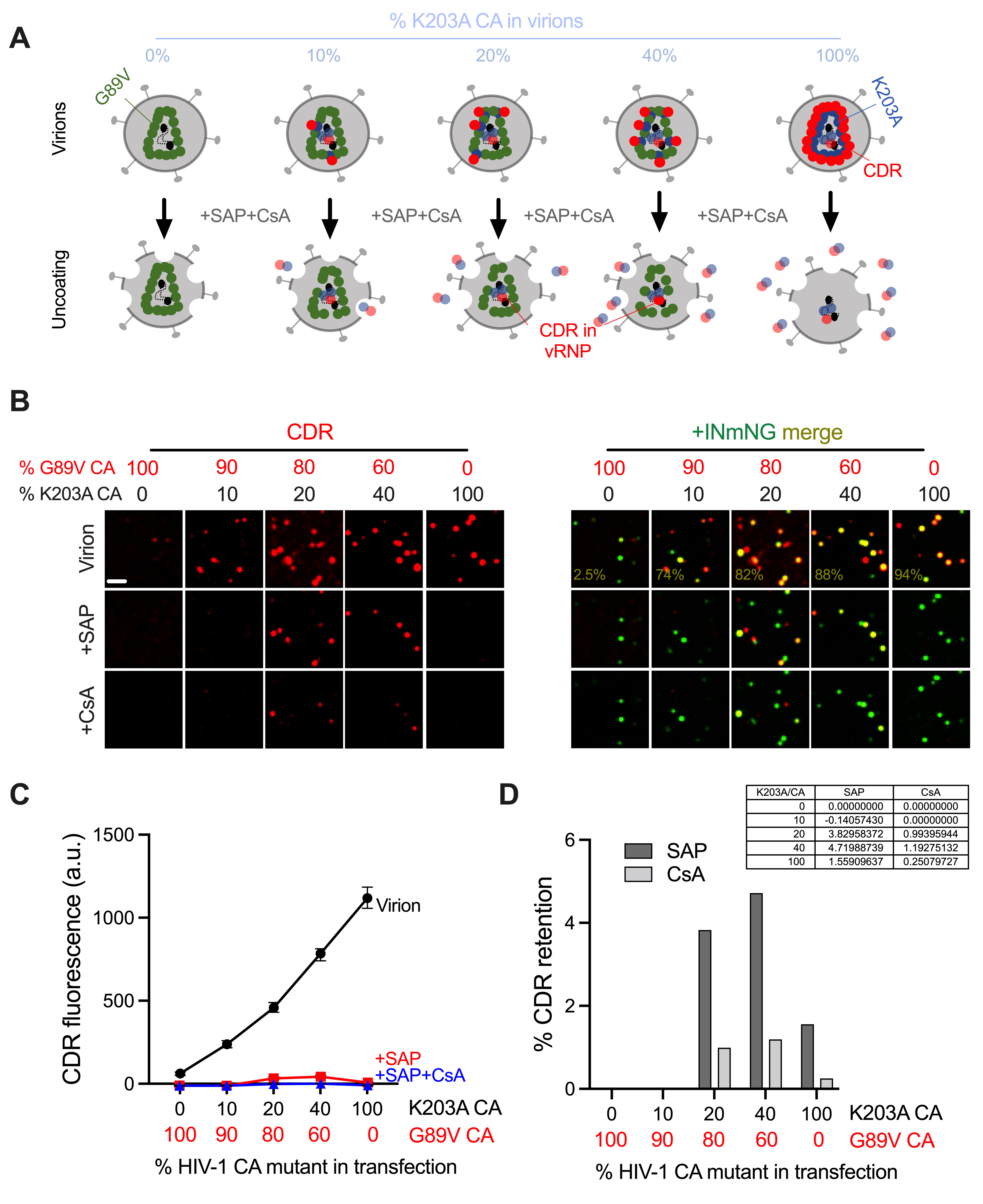

Supplement: S5 Fig — Chimeric HIV-1 pseudovirus with a mixed composition of K203A/G89V capsid were produced by co-transfecting 293T cells with indicated proportions of pR9ΔEnv plasmids encoding for K203A or G89V CA, along with constructs expressing fluorescent INmNG and CDR labels. Single virus particles were bound on poly-l-lysine cover glass and the incorporated CDR-fluorescence was quantitated. (A) Schematics of CDR incorporation into G89V-virions containing an increased proportion of K203A mutant CA, and the release of CDR/CA upon saponin and CsA treatment. (B) Images and (C) fluorescence intensity analysis of CDR incorporation into G89V/K203A mixed CA containing single virions (top panels in B, and black lines in (C)), in cores after saponin treatment (+SAP; middle panel in (B), and red lines in (C)) and in vRNPs after CsA treatment (+CsA; bottom panel in (B), and blue lines in (C)). Images show CDR alone (left panels) and merged with INmNG (green, right panels). Data in (C) is median at 95% confidence interval of sum CDR fluorescence from >1200 single virus particles. Scale bar in (B) is 2 μm. (D) The fraction of CDR fluorescence retained in chimeric cores (+SAP) and in vRNPs (+SAP+CsA) was estimated by normalizing to CDR-fluorescence in 100% K203A/CA virions (set to 100). Normalized values are shown in inset. (TIFF) [file ppat.1010754.s005.tiff]

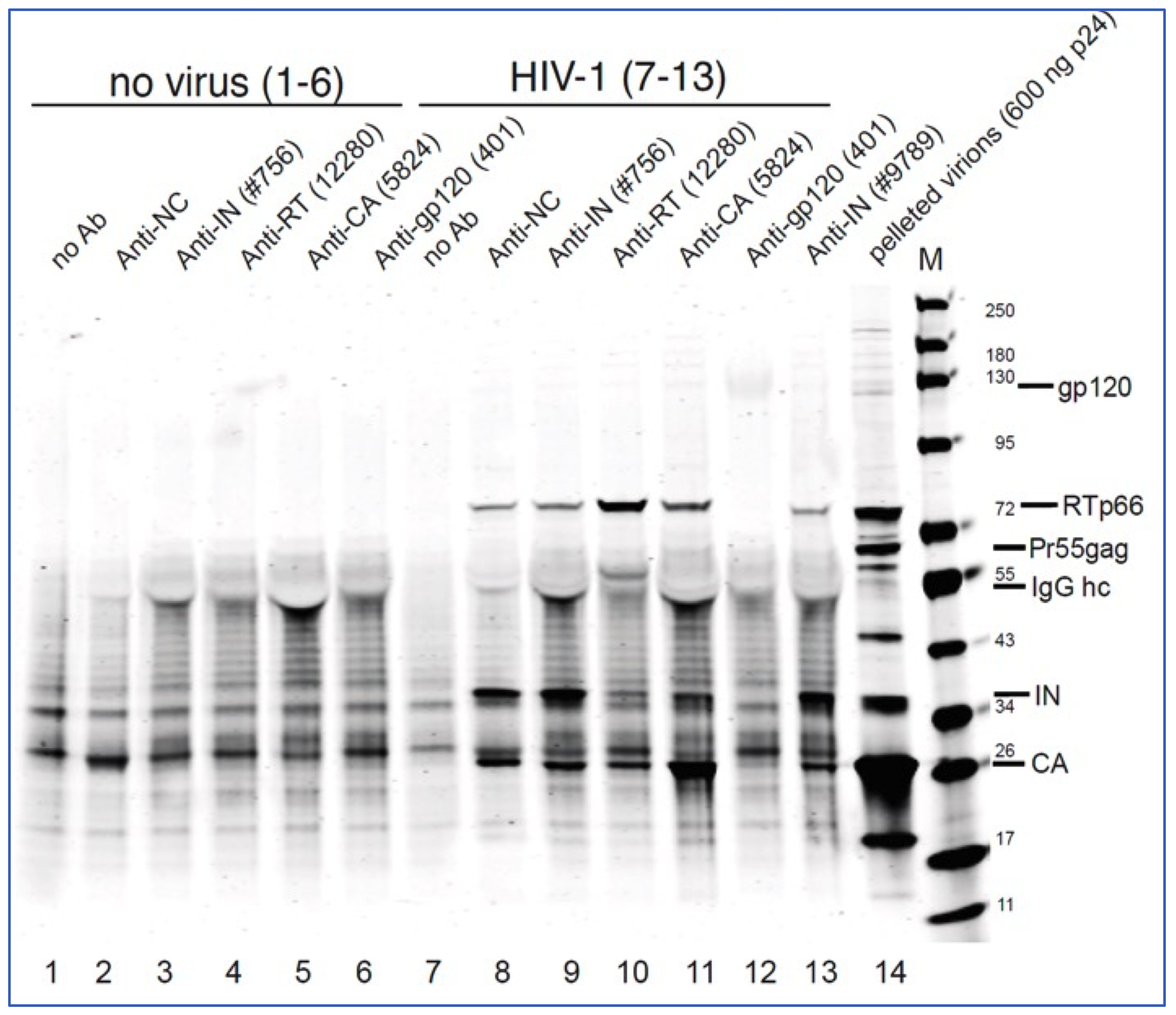

Supplement: S6 Fig — HIV-1 virion lysates were immuno-precipitated using antibodies against vRNP components IN, RT, and NC proteins. Anti-CA and anti-gp120 envelope antibodies were used as controls. Mock immuno-precipitation was done without virus lysate to control for false-positives due to antibody-antibody interactions. The immuno-blot was probed with HIV-IgG human serum (bottom) to identify HIV-1 proteins based on their respective molecular weight. A full immuno-blot relative to the cropped images in Fig 4 is shown. Note the non-specific bands detected in antibody alone control (lanes 1–7) are also detected in the viral lysate IP and migrate differently from distinct RT, IN, NC, CA, and MA proteins that are enriched in lanes with respective antibodies. (TIFF) [file ppat.1010754.s006.tiff]

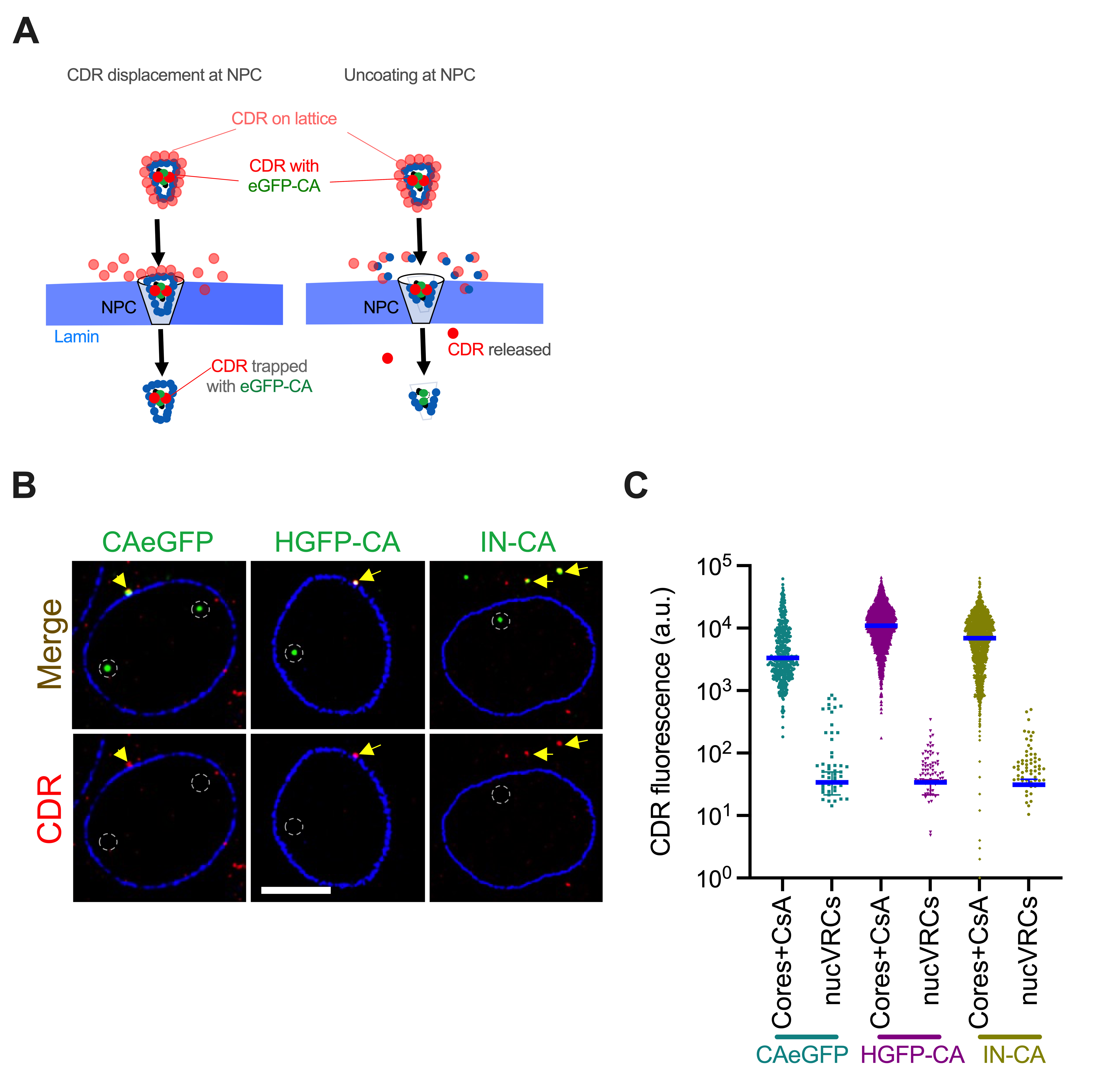

Supplement: S7 Fig — HIV-1 pseudoviruses were co-labeled with CDR (red) along with one of the vRNP associated CA (CAeGFP, HGFP-CA or IN-CA (green)) markers and the retention of eGFP and CDR signals in the nucleus of TZM-bl cells was analyzed. (A) Schematics showing the following scenarios: (1) displacement of CDR from the lattice and entry of intact cores into the nucleus, in this case both eGFP and the vRNP-associated CDR signals should remain colocalized, and (2) Capsid remodeling/ uncoating should result in the loss of high-avidity CDR bound on the lattice and low-affinity CDR bound to eGFP-tagged CA in vRNPs. (B) Single z-slice images showing the detection of HIV-1 labeled with eGFP-tagged CA or INmNG-CA and CDR in the cytoplasm and nucleus of TZM-bl cells at 4 h post infection (MOI 0.2). White dashed lines show CA or IN puncta in the nucleus that does not contain CDR signals and yellow arrow heads point to CDR colocalized with eGFP-tagged CA or IN puncta at the NE and cytoplasm. Scale bars 5 μm. (C) Quantification of CDR signals in single cores upon CsA treatment in vitro and in nuclear VRCs (nucVRCs) at 4 hpi in TZM-bl cells. The retained CDR signal in cores after CsA treatment was used as a comparison for vRNP-associated CDR signals in virions. Data in (C) is median and SEM from 3 independent experiments. Statistics in (C), Mann-Whitney rank sum test. (TIFF) [file ppat.1010754.s007.tiff]

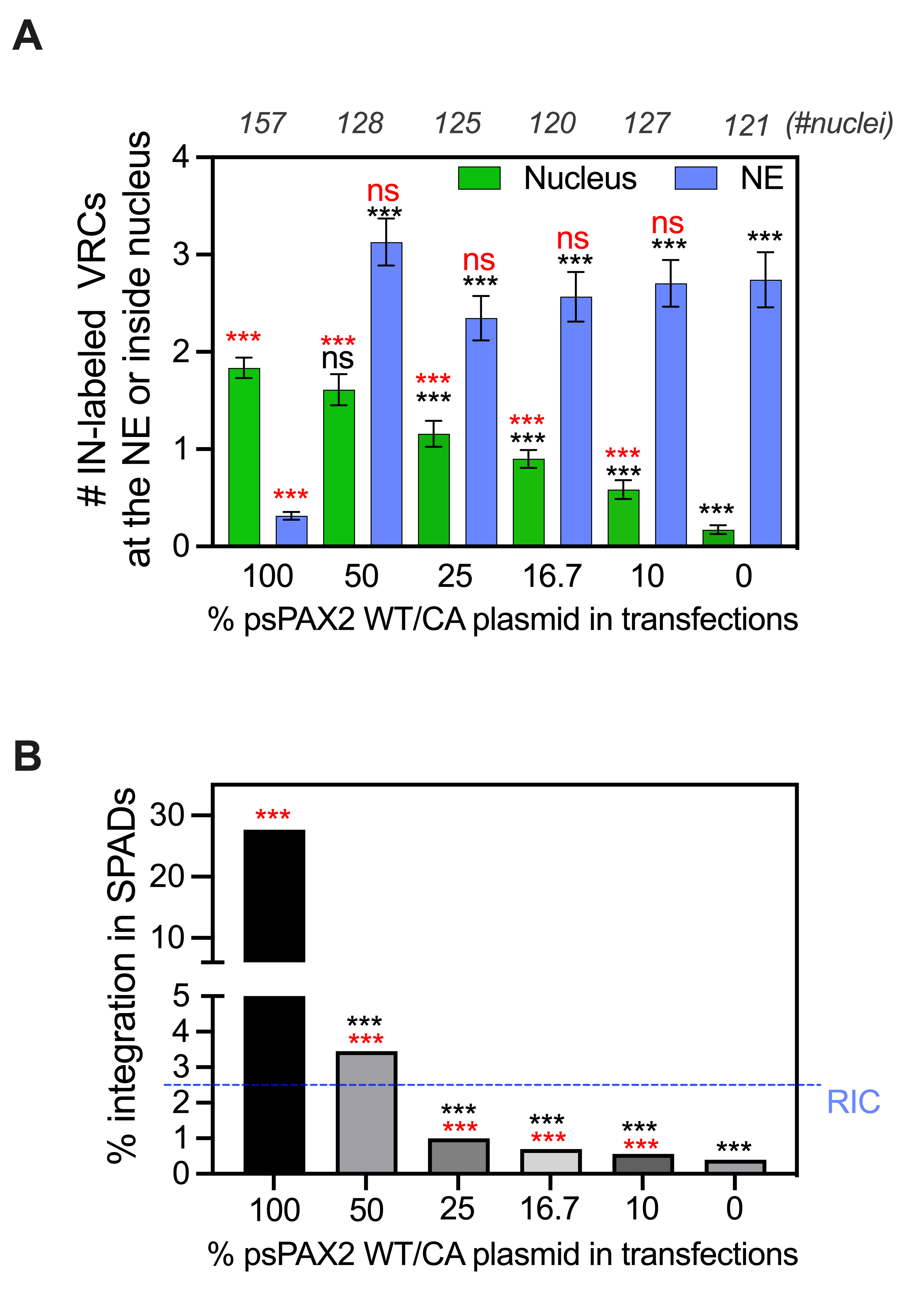

Supplement: S8 Fig — (A) The average number of IN-labeled VRCs inside the nucleus and at the nuclear envelope (NE) was determined in TZM-bl cells at 6 hpi (MOI 0.2) as in Fig 6. (B) HIV-1 integration targeting into SPADs was determined in 293T cells with HIV-1 viruses containing 100% WT or N74D or a indicated mixture of WT and N74D mutant CA. Genomic DNA was extracted 120 hpi and % SPAD integration targeting was determined. The in silico simulated random integration control (RIC) was 2.5% and is shown as dashed blue line. Statistics: Black vs 100% WT CA and in Red vs 100% N74D CA. Data is mean from 2 independent experiments. (TIFF) [file ppat.1010754.s008.tiff]

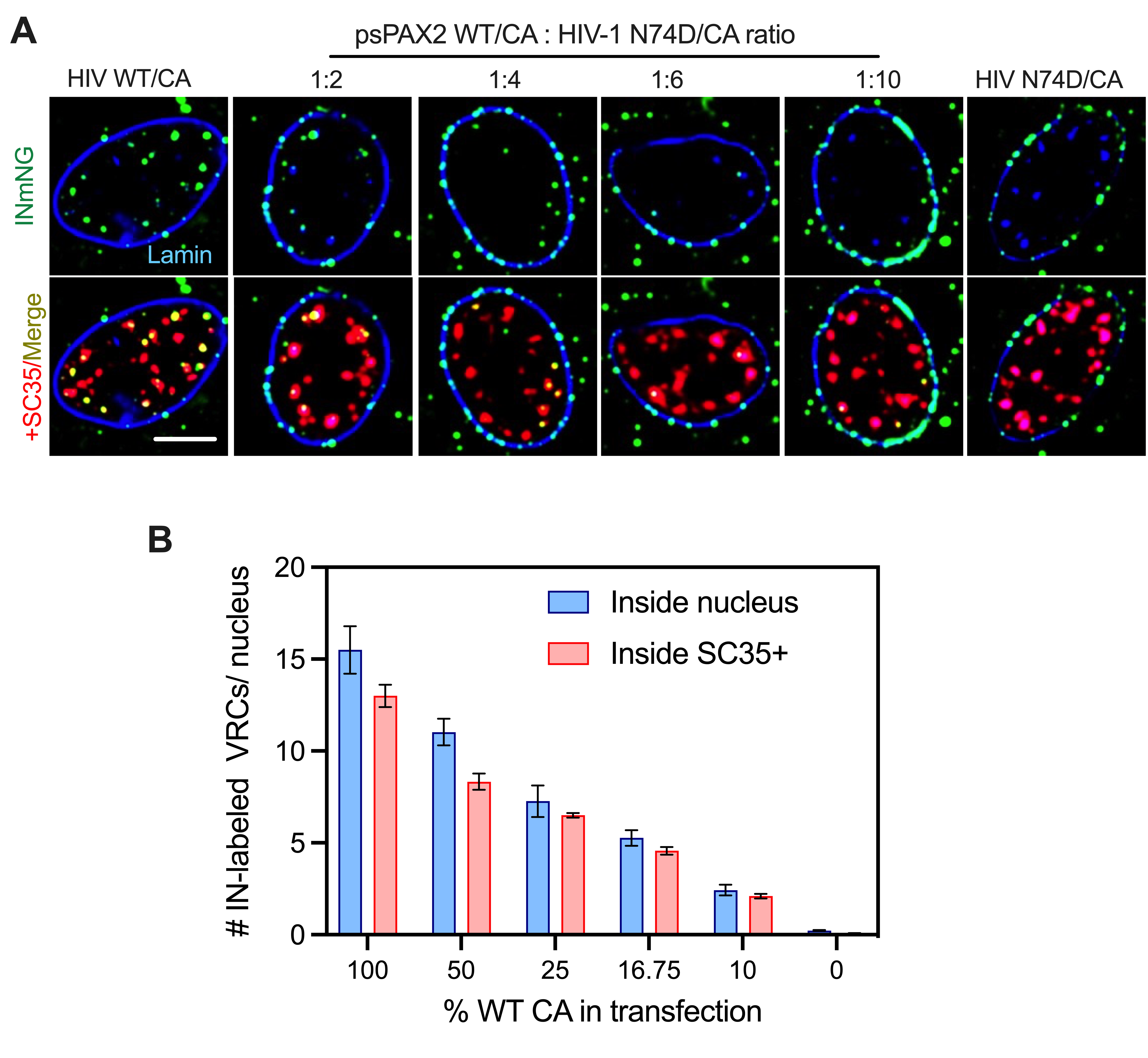

Supplement: S9 Fig — (A) Single Z-plane images showing IN-labeled HIV-1 VRCs (green puncta) colocalized with SC35-nuclear speckles (red) and nuclear envelope (NE)-proximal VRCs in fixed TZM-bl cells at 4 hpi (MOI 2). Note, SC35-nuclear speckles (red) were detected by immunostaining and show borderline overlap with nuclear lamin (blue) Cy5 fluorescence. Scale bar is 5 μm. (B) Quantification of nuclear WT/N74D CA VRCs detected inside >0.5 μm of the NE and inside SC35+ nuclear speckles. The ratio of psPAX2-WT CA: pHIV-N74D CA backbone plasmids in 293T transfections are shown above panels in (A), and the respective proportions are shown in x-axis of (B). Note the 100 and 0% WT/CA in (B) corresponds to viruses produced respectively with pHIV-WT CA and pHIV-N74D CA backbones. Data is mean and SEM from 1 of 2 independent experiments, n>120 nuclei analyzed for each condition. (TIFF) [file ppat.1010754.s009.tiff]

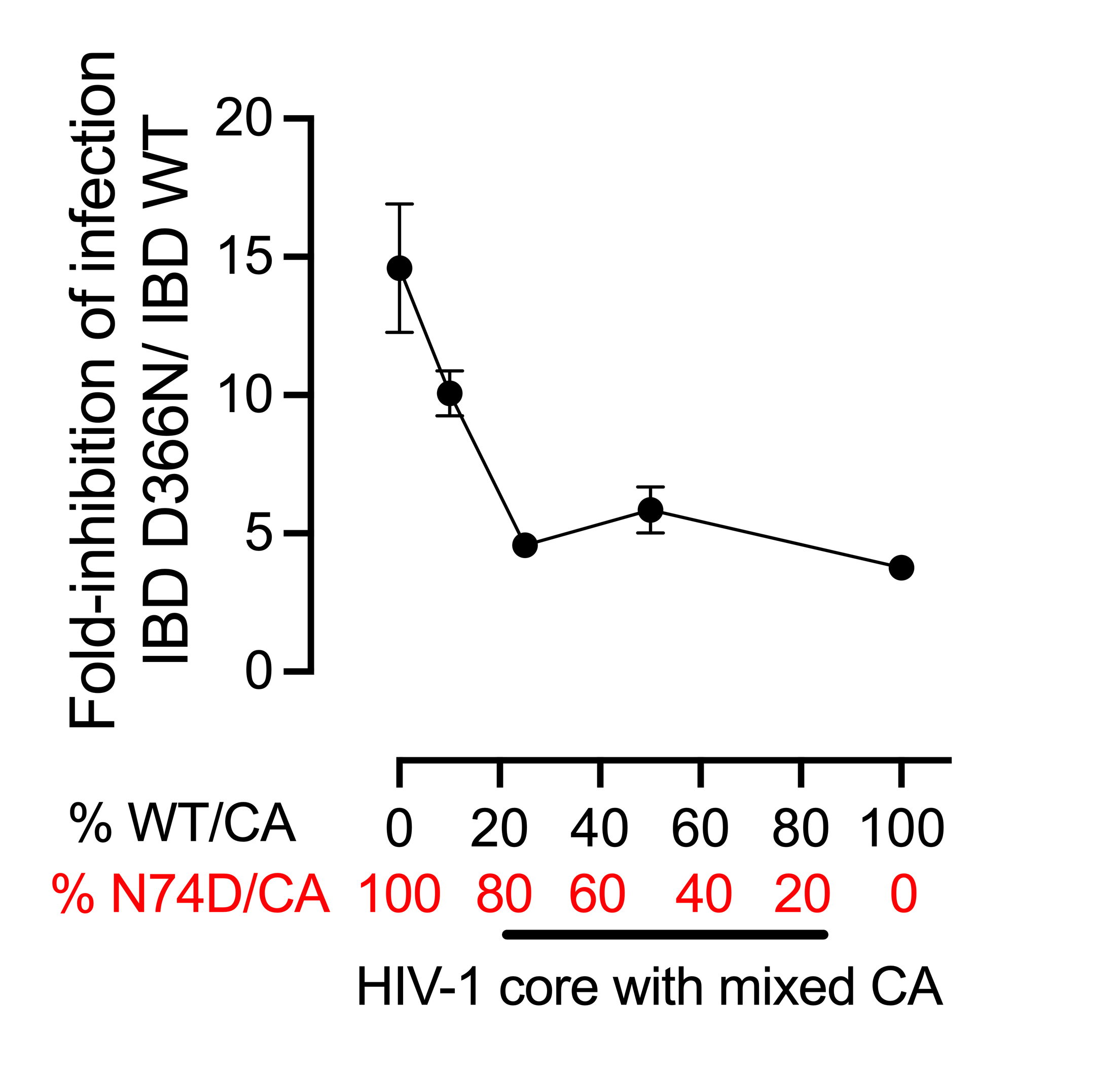

Supplement: S10 Fig — Fold-restriction to single round infectivity of HIV-1 virus with mixed N74D and WT CA proteins suggests an increased restriction of N74D CA containing viruses that remain at the NE. Infectivity of VSV-G pseudotyped HIV-1 containing 100% WT or N74D CA and indicated mixture of N74D/WT CA were analyzed at 72 h in MT4-cells stably expressing NETC-GFP-IBD WT or IBD-D366N mutant protein. Fold-restriction was calculated by dividing the luciferase RLU from IBD-D366N expressing cells from IBD-WT restriction competent cells. Error bars are standard deviation, from 3 experiments. (TIFF) [file ppat.1010754.s010.tiff]
