# Supplementary material for: Localization and functions of native and eGFP-tagged capsid proteins in HIV-1 particles
Source: PLoS Pathog. 2022 Aug 11;18(8):e1010754. doi: 10.1371/journal.ppat.1010754 (PMC9426931; doi:10.1371/journal.ppat.1010754)
Supplement: Attachment — Submitted filename: Response to reviewers_UPDATED.docx [file ppat.1010754.s013.docx]

Reviewer's Responses to Questions

**Part I – Summary**

***Reviewer #1****: Dr. Francis and colleagues performed an elegant study on eGFP-capsid labeled HIV particles. Ref 7 states that eGFP labeled capsid is incorporated in the virus cone and using these viruses a theory was proposed that HIV uncoating of intact virus particles occurs in the nucleus: Ref 30 however, using a slightly different labeling approach, concluded that eGFP-CA mainly resides within the viral cone and import of these proteins in the nucleus is not indicative of nuclear import of intact cones.*

**RE:** We thank the reviewer for these positive comments.

*In this paper Francis compared the two theories. In a set of experiments, they prove that the interpretation of Ref 30 is correct; eGFP-CA resides within the cone and retaining this fraction in the nucleus does not imply uncoating in the nucleus. A stronger conclusion about this finding is even warranted. The present paper refutes major evidence for a nuclear uncoating event as expressed in Ref 7.*

**RE:** We thank the reviewer for this comment and suggestion. We have performed the suggested experiments and included data showing a lack of IN/CA-disappearance in the presence of Raltegravir in Suppl. Fig. 4F. This result further supports the notion that nuclear disappearance of eGFP-tagged CA occurs during integration and does not directly report uncoating.

***Reviewer #2:*** *When, where and how the HIV core uncoats is highly controversial and has been difficult to study due to the lack of direct methods available. In this manuscript, Francis et al. investigate the incorporation of an eGFP-tagged CA construct recently utilised to research uncoating. Rather than being incorporated into the capsid shell of HIV, they find that eGFP-CA is excluded from the shell and instead associates with the vRNP. This suggests that eGFP-CA is not a good tool to study uncoating and questions the interpretation of experimental data acquired using this construct. This is an important finding to alert the field to and is likely to promote much further discussion. In addition, Francis et al. use various mutants to further address uncoating and agree with other recent papers that capsid remodelling is the predominant pathway for nuclear entry.*

**RE:** We thank the reviewer for these positive comments.

*In general, this topic is of great interest in the field and is likely to be relevant to many researchers. The introduction is well written and gives a good account of the current ideas. Unfortunately, the results section is dense and hard to read partly because of the various construct names and partly due to the complex experimental set ups. The authors have obviously tried to address this by including explanatory diagrams in the figures but perhaps it would help to include a few more sentences summarising what the previous section means in more general/overview terms and what the potential outcomes of the next set of experiments could show (ie how the experiment will address different possible scenarios)? Many of the experiments rely on assumptions about what individual techniques are measuring, so although the authors debunk one tool for studying uncoating, they reply on other tools that also have caveats. This is a general limitation of the field but the authors should take care to list and consider alternative explanations for their data, particularly in the discussion section.*

**RE:** We apologize for the dense writing style. We have now significantly modified the manuscript to improve the clarity and facilitate flow. These changes are extensive and can be seen in the mark-up document with tracked changes. We also included additional discussion sections to provide an alternative interpretation of our results. Pg.13 lines 22-31, Pg. 14 lines 1-10, lines 38-45, and Pg. 15, lines 1-7.

*Annoyingly, there are no line numbers!*

**RE:** We apologize for this omission; line numbers are now included.

***Reviewer #3****: This is an interesting study by a collaborative group, which looks into the distribution of the HIV-1 CA protein in the virion. CA is canonically known to make up the capsid shell that surrounds and protects the viral core vRNP particle, which contains the genome and associated replication enzymes. The authors report evidence that some CA is associated with the vRNPs; this is something that has been alluded to before, but seems established here with sufficient rigor.*

**RE:** We appreciate the reviewer’s positive comments.

*To this reviewer, the most significant finding here is that some proportion of CA is associated with the vRNPs, and enters the nucleus as “cargo” rather than as part of the capsid. Unfortunately, this finding is incremental, because it has been suggested before and the authors do not go significantly beyond the phenomenology to elucidate what the vRNP-associated CA is doing. The final set of experiments showing that nuclear transport is sensitive to changes in CA composition is intriguing - WT CA doped into N74D capsid is shown to confer restriction to CPSF6-358 at 10% level, but cannot rescue nuclear import at 50% level - but of unclear significance, as no follow-up is done to determine whether the effect is mediated by capsid-associated or vRNP-associated CA.*

**RE:** We are not aware of papers suggesting that CA interacts with vRNPs or show co-IP of these components, but would be happy to cite these, if such information becomes available.

As suggested by the reviewer, we have done follow-up experiments to test if vRNP localized INmNG-CA (WT) fusion protein (shown in Suppl. Fig. 4) can rescue CPSF6 interactions of the N74D virus. Integration site sequencing analysis reveal that vRNP-localized INmNG-fused CA failed to re-target the N74D virus integration into SPADs, which is now summarized in (Suppl. Table 2). Our results suggest a role for the assembled CA lattice in CPSF6 interactions and nuclear transport (Fig. 2, Fig. 6, Suppl. Figs. 2, 8 and 9). These results are now discussed in pg. 14, lines 38-45, and pg. 15, lines 1-7)

“As an initial test of vRNP-associated CA functionality, we incorporated wild-type CA at the C-terminus of Vpr-INmNG (Vpr-INmNG-CA, Suppl. Fig. 4) and assessed the ability of this novel fusion protein to direct N74D virus integration into SPADs. As shown in (Suppl. Table-2), the basal levels of SPAD targeted integration for the unlabeled WT and N74D viruses was 29.7% and 0.7%, respectively, and these levels remained largely unaffected by the presence of vRNP-associated INmNG-CA proteins. These results suggest the inability of INmNG-CA proteins to associate with CPSF6 and mediate integration targeting. There could be several reasons why CA fused to INmNG failed to appreciably stimulate SPAD-tropic HIV-1 integration targeting (Suppl. Table-2). While CA hexamerizes to form the intra-hexamer CPSF6 binding pocket, functionally relevant IN assemblies are four-fold in nature (tetramer and hexadecamer [1-3]). Thus, the propensity for IN to tetramerize could negate the ability of fused CA moieties to functionally hexamerize. Otherwise, CA may need to be part of the mature capsid lattice to effectively engage CPSF6 for integration site targeting. We surmise that this assembled CA which interacts with CPSF6 in the nucleus originates from a subset of the CA lattice that remains following progressive disassembly/ remodeling of the capsid during virus ingress and nuclear transport [4, 5].”

These results only suggest that INmNG-CA fusion proteins do not form the CPSF6 binding interface, but do not rule out the possibility for untagged-CA in vRNPs to form assemblies capable of CPSF6 interactions. Experiments probing CA assembly in vRNP will require more involved techniques, like cryo-EM, which is beyond the scope of the current study.

*The second aspect of the paper is the claim that eGFP-labeled CA is excluded from the capsid. This is a distinct question (eGFP-CA can be associated with the vRNP without necessarily being excluded from the capsid). The data presented do not rigorously establish this. While somewhat of academic interest, this is nevertheless an important issue because eGFP labeling is now being used as a reporter of uncoating.*

**RE:** We thank the reviewer for this comment. In Fig. 3 we show: (1) loss of eGFP-tagged CA from permeabilized viruses *in vitro* does not correlate with the core stability, and (2) when compared with HIV-1 WT CA cores, the same fraction of eGFP-tagged CA is retained in vRNPs of the unstable K203A CA mutant. These results clearly demonstrate that the majority of the core-associated eGFP-signals are derived from the vRNP-associated fraction of tagged CA, which is likely driven by stable CA-vRNP interactions, as supported by our co-IP data (Fig. 4).

As suggested by the reviewer, we cannot rule out the possibility that a small subset of eGFP-tagged CA is both associated with vRNPs and incorporated in the capsid lattice. However, in this scenario, it will be difficult to distinguish the loss of a few eGFP-signals on the capsid (uncoating) from that associated with vRNPs. We have now discussed this possibility and limitation to interpret GFP loss as uncoating in the Discussion (pg. 13, lines 22-26).

“While our data (Fig. 3) suggests that the majority of core-incorporated eGFP-tagged CA is vRNP associated in unstable K203A capsids, it does not rule out the possibility that a small sub-fraction of tagged-CA is incorporated into the assembled WT capsid. However, in that case, visualizing a complete loss of eGFP-signal in the nucleus is unlikely to reflect capsid uncoating, as these signals would be dominated by the majority of eGFP-tagged CA in the vRNP.”

We maintain that our results showing the propensity of eGFP-CA to associate with vRNPs and its failure to support interactions with capsid-binding host factors demonstrate the failure of this label to accurately report on HIV-1 uncoating.

*The paper will be significantly improved by having a clear mechanistic focus - either on vRNP-associated CA or on capsid-associated CA; as it stands, I think the impact is quite modest.*

**RE:** Our results clearly suggest a role for the assembled CA in CPSF6 interactions and nuclear transport (Fig. 2, Fig. 6, Suppl. Figs. 2, 8 and 9). Additionally, we have now added new data (Suppl. Table 2) showing that vRNP-localized INmNG-CA fusion protein does not contribute to CPSF6-mediated integration targeting in the nucleus. We propose that the subset of CA retained in the nucleus is likely derived from “a subset of the CA lattice which remains following progressing disassembly/ remodeling of the capsid” in pg. 15, lines 6-7.

The structure and function(s) of unlabeled CA inside vRNPs remain unknown. However, our observation that capsids become permeabilized during nuclear import (Fig. 5 and New Suppl. Fig. 7), and CA can interact with vRNPs (Fig. 4), respectively suggests that (1) capsid remodeling is the predominant pathway to nuclear import, and (2) highlights a basis to retain partial CA lattice by VRCs in the nucleus. Which is significant to the field, and we have discussed this in pg. 14, lines 24-37; and pg. 15 line 8-11.

**Part II – Major Issues: Key Experiments Required for Acceptance**

***Reviewer #1****: Unfortunately, few studies were done in this manuscript on the functionality of nuclear complexes containing eGFP-CA. The loss of fluorescence intensity together with IN is taking place at late time points 12 hr, well beyond expected time of integration. When do IN complexes without capsid disappear? What is the location of IN complexes without eGFP-CA? What happens if integration is blocked by integrase inhibitors? This control experiment should be done.*

**RE:** We and others have determined the kinetics of reverse transcription and integration in TZM-bl cells by a time-of-drug-addition assay [6-8]. In these experiments, the half-time of integration in TZM-bl cells is ~12 h, which strongly correlates with the kinetics of nuclear disappearance of IN-labeled HIV-1 replication complexes [6]. We have further demonstrated that the disappearance of nuclear HIV-1 complexes can be efficiently blocked by treatment with raltegravir (RAL) [6, 7]. Similar effects have also been documented by other labs for HIV-1 [9, 10] and also for MLV integration [11].

We have observed the disappearance of IN-labeled complexes as early as 6 h and as late as 20 h post-infection. The timing of the current INmCherry/CAeGFP disappearance is thus well within this window, which we assume to represent *bona fide* integration events. We have now performed additional experiments and include data showing persistence of eGFP-tagged CA/INmCherry labeled complexes in nuclei during Raltegravir treatment (Suppl. Fig. 3F). This result is described in (pg. 8, lines 34-39):

“Moreover, the disappearance of nuclear eGFP-tagged CA/INsfCherry complexes, which was observed in over 22% of cells in control infections, was largely reduced (<5%) upon inhibition of integration with 10 µM raltegravir (+RAL, Suppl. Fig. 3F). A strong correlation between nuclear VRC disappearance and eGFP reporter expression in control cells contrasts with the lack of disappearance in RAL treated cells. These results suggest that eGFP-tagged CA and IN fluorescent signals disappear near simultaneously, likely at the time of functional integration.”

***Reviewer #2****: 1) Figure 3: This experiment should be repeated with the G89V mutant used in Supp Figure 4 in combination with the K203A mutation so that only the trapped CA is labelled with CDR. This would give a better indication of the amount of CA inside the core and when it is released, by SAP and CsA, which is an integral part of this paper.*

**RE:** We thank the reviewer for this idea. We have now performed this experiment and included the results in Suppl. Fig. 5. These results show that CDR incorporation into G89V CA mutant virus (G89V CA does not interact with CDR [12, 13]) is correlated to the amount of K203A mutant CA with an intact CypA-binding loop packaged into chimeric G89V/K203A CA containing virions. Addition of saponin and CsA, revealed low fraction (~1%) of virus incorporated CDR to be retained in K203A CA containing vRNPs. The dependence of CDR incorporation on amino acid G89 of K203A mutant CA suggests that the retained CDR is associated with CA. Moreover, because one molecule of CDR binds to two CA [12, 14], we estimate the fraction of unlabeled CA in vRNPs to account for ~2% of virus incorporated CA. We have added pertinent information under the new section in pg. 10 line 13-39:

**“A fraction of unlabeled CA and CDR gets packaged with vRNPs**

Our results (Fig. 3 and Suppl. Fig. 4) suggest that analysis of CDR retained by cores in the presence of CsA can faithfully report the packaging of tagged CA with vRNPs inside capsids. We next asked if unlabeled CA can also associate with vRNPs, and if this quantity can be estimated by incorporating CDR. To test this possibility, we incorporated INmNG vRNP-marker and CDR into chimeric HIV-1 capsids containing varied proportions of G89V and K203A CA mutants. Virions made by co-transfecting WT and CA mutant constructs were expected to recruit CDR in proportion to the intact CypA-binding loop (amino acids G89, P90) of the K203A CA mutant in the capsids. We reasoned that, following saponin and CsA treatment, the majority of virus-incorporated CDR will be released from capsids containing the unstable K203A CA, and only a subset of CDR will remain bound to unlabeled CA (if any) in the vRNPs (Suppl. Fig. 5A).

Imaging of single virus particles immobilized on poly-L-lysine treated glass revealed that, as expected, virions that contained G89V CA alone failed to efficiently incorporate CDR and showed marginal CDR colocalization (~2.5%) with the INmNG labeled cores. In contrast, mixed G89V/K203A CA virions efficiently incorporated CDR, showing 74-94% colocalization with INmNG labeled cores (Suppl. Fig. 5B). The dependency of CDR on the CypA-binding loop of K203A CA for its incorporation into mixed G89V/K203A capsids was further confirmed by fluorescence intensity analysis, which showed a correlated increase in virion-incorporated CDR fluorescence upon increasing the proportion of K203A CA plasmid in the transfection mixtures (Suppl. Fig. 5C). Addition of saponin released the majority of virus-incorporated CDR from the unstable mixed G89V/K203A capsids, and CsA-treatment released the remaining low-level of CDR from K203A vRNP complexes to near background levels (Suppl. Fig. 5C). Quantitation of the INmNG-associated CDR signals following saponin and CsA treatment relative to the total CDR incorporated into 100% K203A CA-containing virions revealed that as much as ~1% of virion-incorporated CDR (Suppl. Fig. 5D) can be incorporated into vRNPs. Based on the observation that one CDR molecule interacts with two adjacent CA molecules [14], we propose that as much as ~2% of virus incorporated CA is packaged in vRNPs. ”

*2) Related to point1, Supp Figure 6: Why does the GFP signal remain when the CDR signal is lost upon nuclear entry? I believe there is evidence that CypA binds monomeric CA tightly, so the authors explanation of “strong binding to cores and weak binding to monomeric CA” is not correct. Therefore, in the absence of CsA, CypA should remain bound to CA. Perhaps combining this result with Fig3 suggests that the CDR does not actually bind the GFP-tagged CA at all, but some CDR just gets trapped into cores passively. This is then released when cores uncoat? I think the authors make a few too many assumptions here about how their CDR works and so miss other potential explanations?*

**RE:** We are unaware of the high-affinity CypA binding to monomeric CA. Monomeric CypA is known to bind to monomeric CA with low affinity (~10 µM) [15, 16] and with a somewhat higher affinity (~1 µM) to the assembled CA lattice *in vitro* [17]. Accordingly, we and others have demonstrated a poor incorporation of the monomeric labeled-CypA into assembled HIV-1 particles [12, 18]. We have also shown that monomeric CypA is readily released from virions upon membrane permeabilization further supporting its low-affinity binding to capsid. By contrast, the tetrameric CDR tightly binds to the assembling Gag/GagPol precursors and to mature CA lattice (K_D_ < 25nM), and is released only upon loss of CA *in vitro,* and in the cytoplasm of living cells.

Note that, the CDR can be efficiently incorporated into vRNPs of G89V virions *via* an INmNG-CA fusion protein, but not with INmNG alone, suggesting incorporation is CA dependent (Suppl. Fig. 4). Moreover, following this reviewer’s suggestion, we now show that the quantity of virus-incorporated CDR is a linear function of the G89V/K203A CA plasmid ratio (Suppl. Fig. 5).

We show that, unlike binding to the HIV-1 core, CDR does not bind tightly to vRNP-associated eGFP-tagged CA, as evidenced by its release upon uncoating of K203A cores (Fig. 3). Therefore, upon capsid remodeling, we expect that the loosely bound CDR from internal vRNP-associated eGFP-tagged CA to be released. We now include a cartoon in Suppl. Fig. 7A to illustrate this principle. The lack of CDR inside the nucleus in these situations (eGFP-tagged CA or INmNG-CA) likely reflects the permeabilization of capsids during nuclear import. These results are discussed in pg.9, lines 15-24; pg.11, lines 14-31, pg. 14, lines 1-10.

*3) Figure 4: I’m not sure of the validity of the conclusions from the IP experiments done here. Only a fraction of core proteins are pulled down which could be incompletely lysed particles. It seems more likely that all the core proteins come down because the authors are pulling down at least partially intact cores?*

**RE:** HIV-1 vRNP is composed of vRNA, NC, IN, RT, and Vpr [19, 20]. Here we demonstrate that pulldown of any of these proteins brings down the other vRNP components. Importantly, the vRNP-complex is highly resistant to detergents used in IP-experiments, in other words they cannot be disassembled easily *in vitro* and this is likely to explain why multiple vRNP components are IP’ed with IN. We agree with the reviewer that partial capsids can be co-immunoprecipitated with IN and RT. This interpretation additionally supports our notion that assembled CA is retained *via* vRNP interactions in the nucleus.

*By what mechanism do the authors suggest that IN could IP RT and vice versa, as they bind different nucleic acids? Importantly, these particles have not reverse transcribed yet (I believe they are just viral particles in vitro), so there is no vDNA? This is the only evidence the authors can provide to explain how CA could stay associated with IN after uncoating, but it is extremely weak.*

**RE:** It has been shown that IN interacts with HIV-1 genomic RNA [21-23] and that this interaction is essential for proper virus maturation and vRNP formation. Thus, IN and RT can interact with the same form of nucleic acid in virions (the viral genomic RNA). It is also well established that the vRNA genome acts as a scaffold for vRNP formation and this could increase the stability of vRNP complex and explain co-IP of RT and IN. An alternative explanation for co-IP of IN and RT could involve direct interactions between these proteins, as reported previously [24, 25]. Our co-IP data thus shows a potential for CA-vRNP interactions. While our data support one of the mechanisms for retaining a partial CA lattice (*via* CA-vRNP interactions), we are unaware of other means to show direct interactions between CA and vRNP components. We believe this new insight is critical to move the field forward towards a better understanding of uncoating and CA retention in the nucleus. Future work, beyond the scope of this manuscript, will be needed to address which CA residues and partner vRNP components are involved in interactions and retention in the nucleus.

***Reviewer #3****: (1) The authors report a provocative result in Fig 2, in which co-incorporated CA (10%) is tested for its ability to render a capsid made of 90% resistant CA (N74D) susceptible to CPSF6 restriction. A similar experiment is done in Suppl Fig 2, with CypA. The authors conclude, from the differences in behavior of the WT CA-doped vs tagged CA-doped cores that eGFP-labeled CA is excluded from the capsid lattice. This is a critical experiment, but the authors do not show the minimum amount WT CA needs to be incorporated into the mutant capsid for it to be susceptible to restriction. That number estimates the upper limit of incorporation of eGFP-labeled CA into the capsid that would give a false negative in their assays.*

**RE:** This is a good point. In addition to varying 10-50% ratios shown in Fig. 6A, we now show that N74D viruses containing 5% of unlabeled WT CA (new Fig. 6A) are recognized and restricted by CPSF6-358 in the cytoplasm. While we did not explore lower ratios of WT/N74D CA, viral particles containing <5% of eGFP-tagged CA cannot be detected by live cell fluorescence microscopy and are thus not amenable to single virus uncoating studies.

We would like to point out that, the same low levels of eGFP-CA were retained by vRNPs following saponin treatment of unstable K203A capsids and WT capsids (Fig. 3). This result suggests that the majority of core associated eGFP-signal resides within vRNPs. However, we cannot rule out the possibility that a miniscule fraction of eGFP-CA can still co-assembled with unlabeled WT CA into a capsid lattice. We now discuss this alternative scenario in pg. 13, lines 22-26.

**“**While our data (Fig. 3) suggests that the majority of core-incorporated eGFP-tagged CA is vRNP associated in unstable K203A capsids, it does not rule out the possibility that a small sub-fraction of tagged-CA is incorporated into the assembled WT capsid. However, in that case, visualizing a complete loss of eGFP-signal in the nucleus is unlikely to reflect capsid uncoating, as these signals would be dominated by the majority of eGFP-tagged CA in the vRNP. ”

*(2) Page 9: Experiments reported here can be explained by a simple model in which passage through the nuclear pore channel strips the bound CDR from the capsid surface, even if the capsid remains fully intact. The claim that the capsid undergoes remodeling or is permeabilized during import may be true, but is not rigorously established by the presented data. More evidence is needed.*

**RE:** We agree - it is possible that CDR is stripped during nuclear import. To test this possibility, we re-directed a subset of CDR to vRNPs by eGFP-tagged CA or by incorporating INmNG-CA fusion protein (Suppl. Fig. 4 and 6) and asked if the internal CDR is retained in the nucleus. We now include a cartoon (Suppl. Fig. 7A) to illustrate a loss and/or retention of vRNP-associated CDR inside the nucleus, by capsid remodeling and passage of intact capsids into the nucleus, respectively. We reasoned that, if the intact core can transit through the nuclear pore, CDR loosely associated with the vRNP would be retained and be detectable inside the nucleus. However, as shown in (Suppl. Fig. 7B, C), the CDR signals in the nucleus is significantly (>1-log) lower than those inside immobilized cores on glass, suggesting vRNP associated CDR is lost during nuclear import and supporting capsid remodeling/permeabilization. It has also been shown [10] that IN- in pre-integration complexes can also be remodeled during nuclear import. Perhaps this re-modelling of IN in vRNPs can additionally account for loss of the trapped vRNP-associated CDR. We discuss this in pg.14, lines 5-10.

**“**Based on these new results, we propose that, in addition to compositional changes during HIV-1 nuclear import, remodeling of the CA lattice will likely involve local rearrangements (structural changes), resulting in a loss of avid CDR-CA interactions at the NPC [7]. Loss of CDR from eGFP-CA and INmNG-CA tagged VRCs in the nucleus may be explained by dynamic changes in IN oligomerization observed during HIV-1 nuclear import [10].”

For rigor, we also established a different approach to test capsid permeabilization. We show that IN inside VRCs becomes exposed to chimeric integrase binding domains (IBD) at the nuclear pore (Fig. 5). We also show (Fig. 6) the marked differences in capsid interactions with the cytoplasmic and nuclear CPSF6 forms. Together, these results imply that capsids become permeabilized and remodeled during nuclear import. We are unaware of other methods capable of reporting capsid remodeling than what is already published and what is in this paper.

**Part III – Minor Issues: Editorial and Data Presentation Modifications**

Please use this section for editorial suggestions as well as relatively minor modifications of existing data that would enhance clarity.

***Reviewer #1****: 1. Abstract; eGFP-CA is not a relevant marker for uncoating; replace by is not a “direct” marker of uncoating, it may be used as an indirect marker (some loss during nuclear import, altered accessibility)*

**RE:** we have corrected the abstract, it now reads “These results thus imply that eGFP-tagged CA is not a direct marker for capsid uncoating.”

*2. Intro: the statement that HIV intranuclear transport is determined by CA-CSPF6 interaction should be rephrased taking the results of this paper into account. Does intranuclear CA-CPSF6 interaction relate to cone capsid or within cone capsid?*

**RE:** we now address this question in the introduction (pg. 4, lines 36-43):

*Is there any evidence that capsid-IN complexes in the nucleus are functional?*

**RE:** While we cannot be sure if all CAeGFP/IN complexes are functional, the co-disappearance of eGFP-tagged CA and INmCherry during integration for the tracked VRC subset suggests that at least a fraction of the nuclear eGFP-tagged CA complexes is functional. This point is now more rigorously established by the block of disappearance using Raltegravir (Suppl. Fig. 3F).

*3. Figure 1 A, B add also plasmid eGFP-CA labeling scheme of Ref 30*

**RE:** We have included the design of CAeGFP from Zurnic *et al*. in Suppl. Fig. 1A, and added additional description comparing HGFP-CA and MA-CAeGFP (in pg. 6, lines 9-12).

**“**This labeling approach differs from the recently described N-terminal eGFP-CA fusion protein (Fig. 1B) (hereafter referred to as HGFP-CA, [8]), and C-terminal CAeGFP [9] (Suppl. Fig. 1A), which are expressed in the context of full-length Gag/GagPol.”

*4. Figure 1 G what is the eGFP fluorescence in cytoplasmatic PICs? What is the coloc IN/Capsid in nucleus vs cytoplasma?*

**RE:** This information is now included in the new Figure 1G. We also moved the comparative fluorescence analysis of nuclear VRCs between 4 vs 12 hpi to Suppl. Fig. 1B. Details are included in the figures, and in pg. 6, lines 38-44, and pg. 7, lines 1-11.

*5. P4 Zurnic et al (Ref 30) already concluded that labeled capsid was present within the cone; this manuscript is conforming this result; the finding is a confirmation not an entirely novel finding; this should be added to the intro.*

**RE:** Zurnic *et al*. reported results that were suggestive of CAeGFP being inside cones. Here, we implemented several innovative approaches to rigorously establish that eGFP-tagged CA is packaged with the vRNP inside the core, and that, even unlabeled CA, can interact with vRNP components (co-IP results Fig. 4). Importantly, the localization of CAeGFP with vRNPs or the ability of unlabeled CA to interact with vRNPs has not been shown by Zurnic *et al*.. Our novel findings thus reveal interactions that may potentially retain partial CA lattices in the nucleus.

*6. Can a 1% eGFP-mutant CA contribution to the cones be excluded in the restriction assays?*

**RE:** We cannot rule out this possibility, but, even if such a small fraction of labeled CA does incorporate into capsids, this signal will not be detectable over a background signal by fluorescence microscopy. We now discuss this alternative scenario in pg. 13, lines 22-26.

**“**While our data (Fig. 3) suggests that the majority of core-incorporated eGFP-tagged CA is vRNP associated in unstable K203A capsids, it does not rule out the possibility that a small sub-fraction of tagged-CA is incorporated into the assembled WT capsid. However, in that case, visualizing a complete loss of eGFP-signal in the nucleus is unlikely to reflect capsid uncoating, as these signals would be dominated by the majority of eGFP-tagged CA in the vRNP. ”

***Reviewer #2****: 1) Page 4, last paragraph: The sentence “Since MA-CAeGFP lacks NC and P6, it is incorporated into particles alongside unlabelled CA/Gag” is confusing. Do the authors mean tagged CA is incorporated “by binding unlabelled CA”? More explanation on how this protein is incorporated into both virions and mature cores should be provided.*

**RE:** For clarity, we have now modified the sentence (old text, pg. 4, line 44): “MA-CAeGFP is incorporated into virus particles alongside unlabeled CA/Gag”, as follows in new text pg. 6, lines 2-4:

“We constructed a plasmid encoding HIV-1 MA-CAeGFP fusion protein that is incorporated into virions through MA-driven co-assembly with unlabeled Gag/GagPol polyproteins encoded by the viral backbone (Fig. 1A).”

Further, we now describe the relevance of Vpr-IN co-incorporation, as follows (pg. 6, lines 5-9):

“MA-CAeGFP lacks the nucleocapsid (NC) and p6 domains of Gag. Therefore, co-incorporation of a second fluorescent marker, Vpr-IN fused to super-folder-mCherry (INsfCherry), which is incorporated *via* Vpr interactions with the p6 domain of unlabeled Gag/GagPol [26], was used to identify virions containing a mixture of unlabeled CA and CAeGFP.”

*2) Figure 1: In previous reports (for example studies by the Hope lab) ~50% of Vpr-GFP is lost from particles following membrane fusion. Why is more of the Vpr-IN retained here? Are the authors proposing the Vpr-IN fusion associates with wt IN after maturation so that it is preferentially retained inside the core? As this is the authors control for particles, it would be good to add more explanation of how this marker is behaving.*

**RE:** Yes, the IN-fluorescent proteins construct appears to specifically associate with vRNPs due to their propensity to form higher-order oligomers with unlabeled IN and remain associated with the viral complex through the nuclear import, until integration [4, 6, 7, 9, 10, 12, 27-35]. Since this was not the main focus of the paper, we have summarized this information on pg. 9, lines 40-42:

“INmNG-CA is expected to preferentially package into vRNPs through association with the viral genome and oligomerization with unlabeled IN and, because of this, to recruit CDR to vRNPs [7, 27, 28]. ”

Additionally, as pointed out by the reviewer, the eGFP-Vpr which is packaged with the vRNP within cores [20, 36], dissociates from the core very early in the cytoplasm of cells [36-38]. These results further support the notion that uncoating may be initiated in the cytoplasm.

*3) Figure 1: If the MOI is 0.5, why are there so many dots following the IN or CA staining? Some explanation of what the authors think the dots are is needed.*

**RE:** We agree with the reviewer, that in a fixed cell setting it is virtually impossible to derive functional insights into which particles infect a cell. At a low MOI (0.2), we typically observe 2-4 HIV-1 VRCs in the nucleus and several in the cytoplasm by 4 hpi. Of note, we and others [7, 30] have shown that only ~1 out of 50 incoming viruses enters the nucleus (i.e., the efficiency of nuclear import is ~2%). Furthermore, we have shown that only 1 in 4 HIV-1 complexes in the nucleus disappear during integration and thus contribute to infection [7]. The reasons for this poor efficiency remain unclear. It is likely that steps, such as completion of reverse transcription in the nucleus, might be limiting. The higher number of eGFP-CA and INsfCherry spots in Fig.1 reflects the increased virus input.

*4) Figure 6B-C: These experiments should be done using the CAeGFP construct to determine whether CAeGFP colocalises with the VRC in nuclear speckles.*

**RE:** As per the reviewer’s request, we have now done this experiment and included pertinent data in Suppl. Fig. 9. A higher MOI (MOI 2) was used in this experiment to clearly locate the few HIV-1 complexes from mixed N74D/WT CA viruses inside the nucleoplasm and nuclear speckles. Pertinent information is added in pg. 12, lines 29-31:

“We do note that the few mixed WT/N74D HIV-1 VRCs that penetrated the nucleoplasm efficiently co-localized with SC35-labeled nuclear speckles (Suppl. Fig. 9)..”

*5) Supp Figure 6: Figure legend says 6 hpi but text says 4hpi.*

**RE:** Both the main text, pg. 11, line 25, and related Suppl. Fig. 7 (old Suppl. Fig. 6) legends now state the time as 4 hpi.

*6) I think more deliberation of the experiments in Fig 3, 4 and Supp Fig 6 is needed in the discussion section*

**RE:** We have now added additional discussion points with alternative interpretations for data in Fig. 3, in pg. 13, lines 22-31; Interpretation of data in new suppl. Fig. 7 (old Suppl. Fig. 6) in pg. 14, lines 1-10; Interpretation and implications of data in Fig. 4 are discussed in pg. 14, lines 38-45, and pg. 15, lines 1-16:

***Reviewer #3****: (1) Page 4 – par 2, line 4-5: “did not noticeably affect overall Gag/GagPol cleavage patterns” – this statement is not supported by Suppl. Fig 1, which shows that CA staining is more intense than Gag. Fid 1D, E should include this important control, and the data should be described accurately and interpreted accordingly.*

**RE:** We apologize for the confusion. The thick Gag bands seen in CAeGFP lanes in Suppl. Fig. 1 is an overlap of unlabeled-Gag (pr55 kDa) and CAeGFP (p51 kDa) proteins. The sizes of CAeGFP and MA-CAeGFP are marked. We therefore surmise that the overall Gag processing was not noticeably affected. The old figure marked CAeGFP as 49 kDa, and this is now corrected with the right molecular weight, which is 51 kDa.

*(2) Fig 6A: The authors should test percentages between 10% and 0%.*

**RE:** A new Suppl. Table 1 is included showing the statistical significance and % values of SPAD-targeted integration between all the different concentrations of WT CA tested, against N74D CA virus (0% WT CA).

*(3) Page 4 – par 1, line 5: “N-terminally labeled HGFP-CA and C-terminally labeled CAeGFP”*

**RE:** We have corrected this in pg. 6, lines 13-14, which now reads: “In this study, we compared the N-terminally labeled HGFP-CA and C-terminally labeled CAeGFP labeling approaches to label HIV-1 capsids”

*(4) Page 4 – par 2: Please explain what is being visualized in Fig 1c – it seems these are purified virions, but the text should just say this clearly*

**RE:** Pg. 6, paragraph 2, lines 16-19 now states that analysis in Fig. 1C was done on intact particles.

**“**Analysis of fluorescently tagged intact viral particles bound on poly-L-lysine treated glass”

*(5) Fig 1F – “normalized to unlabeled HIV-1” – please explain in the text or methods how normalization was done*

**RE:** The information regarding the infectivity assay and normalization is now included in Figure 1 legend:

*(6) Page 6 – CPSF6, SEC24C and NUP153 do not interact with “the binding pocket between neighboring hexamers”; the pocket is intra-hexameric*

**RE:** As per reviewers’ suggestions, we corrected the sentence in pg. 7, lines 16-17, as follows: “Other cellular proteins, including CPSF6, SEC24C, and Nup153, interact with the common intra-hexamer CA binding pocket on capsid”

*(7) Please include line numbers to facilitate reviewing and commenting*

**RE**: we apologize for this inconvenience; the line numbers are now added.

**References**

1.	Ballandras-Colas A, Maskell DP, Serrao E, Locke J, Swuec P, Jonsson SR, et al. A supramolecular assembly mediates lentiviral DNA integration. Science. 2017;355(6320):93-5. doi: 10.1126/science.aah7002. PubMed PMID: 28059770; PubMed Central PMCID: PMCPMC5321526.

2.	Passos DO, Li M, Jozwik IK, Zhao XZ, Santos-Martins D, Yang R, et al. Structural basis for strand-transfer inhibitor binding to HIV intasomes. Science. 2020;367(6479):810-4. Epub 20200130. doi: 10.1126/science.aay8015. PubMed PMID: 32001521; PubMed Central PMCID: PMCPMC7357238.

3.	Passos DO, Li M, Yang R, Rebensburg SV, Ghirlando R, Jeon Y, et al. Cryo-EM structures and atomic model of the HIV-1 strand transfer complex intasome. Science. 2017;355(6320):89-92. Epub 2017/01/07. doi: 10.1126/science.aah5163. PubMed PMID: 28059769; PubMed Central PMCID: PMCPMC5508583.

4.	Hulme AE, Kelley Z, Foley D, Hope TJ. Complementary assays reveal a low level of CA associated with nuclear HIV-1 viral complexes. J Virol. 2015. Epub 2015/03/06. doi: 10.1128/JVI.00476-15. PubMed PMID: 25741002.

5.	Campbell EM, Hope TJ. HIV-1 capsid: the multifaceted key player in HIV-1 infection. Nat Rev Microbiol. 2015;13(8):471-83. Epub 2015/07/17. doi: 10.1038/nrmicro3503. PubMed PMID: 26179359.

6.	Francis AC, Marin M, Prellberg MJ, Palermino-Rowland K, Melikyan GB. HIV-1 Uncoating and Nuclear Import Precede the Completion of Reverse Transcription in Cell Lines and in Primary Macrophages. Viruses. 2020;12(11). Epub 2020/11/05. doi: 10.3390/v12111234. PubMed PMID: 33143125; PubMed Central PMCID: PMCPMC7693591.

7.	Francis AC, Melikyan GB. Single HIV-1 Imaging Reveals Progression of Infection through CA-Dependent Steps of Docking at the Nuclear Pore, Uncoating, and Nuclear Transport. Cell Host Microbe. 2018;23(4):536-48 e6. doi: 10.1016/j.chom.2018.03.009. PubMed PMID: 29649444.

8.	Burdick RC, Li C, Munshi M, Rawson JMO, Nagashima K, Hu WS, et al. HIV-1 uncoats in the nucleus near sites of integration. Proc Natl Acad Sci U S A. 2020;117(10):5486-93. Epub 2020/02/26. doi: 10.1073/pnas.1920631117. PubMed PMID: 32094182.

9.	Zurnic Bonisch I, Dirix L, Lemmens V, Borrenberghs D, De Wit F, Vernaillen F, et al. Capsid-Labelled HIV To Investigate the Role of Capsid during Nuclear Import and Integration. J Virol. 2020;94(7). Epub 2020/01/17. doi: 10.1128/JVI.01024-19. PubMed PMID: 31941774; PubMed Central PMCID: PMCPMC7081887.

10.	Borrenberghs D, Dirix L, De Wit F, Rocha S, Blokken J, De Houwer S, et al. Dynamic Oligomerization of Integrase Orchestrates HIV Nuclear Entry. Sci Rep. 2016;6:36485. Epub 2016/11/11. doi: 10.1038/srep36485. PubMed PMID: 27830755; PubMed Central PMCID: PMCPMC5103197 holders on patents related to LEDGF/p75 and LEDGINs. The other authors declare no competing financial interests.

11.	Borrenberghs D, Zurnic I, De Wit F, Acke A, Dirix L, Cereseto A, et al. Post-mitotic BET-induced reshaping of integrase quaternary structure supports wild-type MLV integration. Nucleic Acids Res. 2019;47(3):1195-210. doi: 10.1093/nar/gky1157. PubMed PMID: 30445610; PubMed Central PMCID: PMCPMC6379647.

12.	Francis AC, Marin M, Shi J, Aiken C, Melikyan GB. Time-Resolved Imaging of Single HIV-1 Uncoating In Vitro and in Living Cells. PLoS Pathog. 2016;12(6):e1005709. Epub 2016/06/21. doi: 10.1371/journal.ppat.1005709. PubMed PMID: 27322072; PubMed Central PMCID: PMC4913920.

13.	Luban J, Bossolt KL, Franke EK, Kalpana GV, Goff SP. Human immunodeficiency virus type 1 Gag protein binds to cyclophilins A and B. Cell. 1993;73(6):1067-78. Epub 1993/06/18. PubMed PMID: 8513493.

14.	Ni T, Zhu Y, Yang Z, Xu C, Chaban Y, Nesterova T, et al. Structure of native HIV-1 cores and their interactions with IP6 and CypA. Sci Adv. 2021;7(47):eabj5715. Epub 20211119. doi: 10.1126/sciadv.abj5715. PubMed PMID: 34797722; PubMed Central PMCID: PMCPMC8604400.

15.	Zhou J, Price AJ, Halambage UD, James LC, Aiken C. HIV-1 Resistance to the Capsid-Targeting Inhibitor PF74 Results in Altered Dependence on Host Factors Required for Virus Nuclear Entry. J Virol. 2015;89(17):9068-79. Epub 2015/06/26. doi: 10.1128/JVI.00340-15. PubMed PMID: 26109731; PubMed Central PMCID: PMC4524096.

16.	Schaller T, Ocwieja KE, Rasaiyaah J, Price AJ, Brady TL, Roth SL, et al. HIV-1 capsid-cyclophilin interactions determine nuclear import pathway, integration targeting and replication efficiency. PLoS Pathog. 2011;7(12):e1002439. Epub 2011/12/17. doi: 10.1371/journal.ppat.1002439. PubMed PMID: 22174692; PubMed Central PMCID: PMC3234246.

17.	Marquez CL, Lau D, Walsh J, Shah V, McGuinness C, Wong A, et al. Kinetics of HIV-1 capsid uncoating revealed by single-molecule analysis. Elife. 2018;7. Epub 2018/06/08. doi: 10.7554/eLife.34772. PubMed PMID: 29877795; PubMed Central PMCID: PMCPMC6039174.

18.	Campbell EM, Perez O, Melar M, Hope TJ. Labeling HIV-1 virions with two fluorescent proteins allows identification of virions that have productively entered the target cell. Virology. 2007;360(2):286-93. Epub 2006/11/25. doi: 10.1016/j.virol.2006.10.025. PubMed PMID: 17123568; PubMed Central PMCID: PMC1885464.

19.	Forshey BM, Aiken C. Disassembly of human immunodeficiency virus type 1 cores in vitro reveals association of Nef with the subviral ribonucleoprotein complex. J Virol. 2003;77(7):4409-14. PubMed PMID: 12634398.

20.	Forshey BM, von Schwedler U, Sundquist WI, Aiken C. Formation of a human immunodeficiency virus type 1 core of optimal stability is crucial for viral replication. J Virol. 2002;76(11):5667-77. PubMed PMID: 11991995.

21.	Kutluay SB, Perez-Caballero D, Bieniasz PD. Fates of retroviral core components during unrestricted and TRIM5-restricted infection. PLoS Pathog. 2013;9(3):e1003214. Epub 2013/03/19. doi: 10.1371/journal.ppat.1003214. PubMed PMID: 23505372; PubMed Central PMCID: PMC3591316.

22.	Elliott JL, Eschbach JE, Koneru PC, Li W, Puray-Chavez M, Townsend D, et al. Integrase-RNA interactions underscore the critical role of integrase in HIV-1 virion morphogenesis. Elife. 2020;9. Epub 20200922. doi: 10.7554/eLife.54311. PubMed PMID: 32960169; PubMed Central PMCID: PMCPMC7671690.

23.	Kessl JJ, Kutluay SB, Townsend D, Rebensburg S, Slaughter A, Larue RC, et al. HIV-1 Integrase Binds the Viral RNA Genome and Is Essential during Virion Morphogenesis. Cell. 2016;166(5):1257-68 e12. doi: 10.1016/j.cell.2016.07.044. PubMed PMID: 27565348; PubMed Central PMCID: PMCPMC5003418.

24.	Tekeste SS, Wilkinson TA, Weiner EM, Xu X, Miller JT, Le Grice SF, et al. Interaction between Reverse Transcriptase and Integrase Is Required for Reverse Transcription during HIV-1 Replication. J Virol. 2015;89(23):12058-69. Epub 20150923. doi: 10.1128/JVI.01471-15. PubMed PMID: 26401032; PubMed Central PMCID: PMCPMC4645309.

25.	Hehl EA, Joshi P, Kalpana GV, Prasad VR. Interaction between human immunodeficiency virus type 1 reverse transcriptase and integrase proteins. J Virol. 2004;78(10):5056-67. doi: 10.1128/jvi.78.10.5056-5067.2004. PubMed PMID: 15113887; PubMed Central PMCID: PMCPMC400328.

26.	Bachand F, Yao XJ, Hrimech M, Rougeau N, Cohen EA. Incorporation of Vpr into human immunodeficiency virus type 1 requires a direct interaction with the p6 domain of the p55 gag precursor. J Biol Chem. 1999;274(13):9083-91. Epub 1999/03/20. doi: 10.1074/jbc.274.13.9083. PubMed PMID: 10085158.

27.	Francis AC, Marin M, Singh PK, Achuthan V, Prellberg MJ, Palermino-Rowland K, et al. HIV-1 replication complexes accumulate in nuclear speckles and integrate into speckle-associated genomic domains. Nat Commun. 2020;11(1):3505. Epub 2020/07/16. doi: 10.1038/s41467-020-17256-8. PubMed PMID: 32665593; PubMed Central PMCID: PMCPMC7360574.

28.	Koneru PC, Francis AC, Deng N, Rebensburg SV, Hoyte AC, Lindenberger J, et al. HIV-1 integrase tetramers are the antiviral target of pyridine-based allosteric integrase inhibitors. Elife. 2019;8. Epub 2019/05/24. doi: 10.7554/eLife.46344. PubMed PMID: 31120420; PubMed Central PMCID: PMCPMC6581505.

29.	Francis AC, Di Primio C, Quercioli V, Valentini P, Boll A, Girelli G, et al. Second generation imaging of nuclear/cytoplasmic HIV-1 complexes. AIDS Res Hum Retroviruses. 2014;30(7):717-26. Epub 2014/05/07. doi: 10.1089/AID.2013.0277. PubMed PMID: 24798748; PubMed Central PMCID: PMC4077004.

30.	Burdick RC, Delviks-Frankenberry KA, Chen J, Janaka SK, Sastri J, Hu WS, et al. Dynamics and regulation of nuclear import and nuclear movements of HIV-1 complexes. PLoS Pathog. 2017;13(8):e1006570. Epub 2017/08/23. doi: 10.1371/journal.ppat.1006570. PubMed PMID: 28827840; PubMed Central PMCID: PMCPMC5578721.

31.	Burdick RC, Hu WS, Pathak VK. Nuclear import of APOBEC3F-labeled HIV-1 preintegration complexes. Proc Natl Acad Sci U S A. 2013;110(49):E4780-9. Epub 2013/11/20. doi: 10.1073/pnas.1315996110. PubMed PMID: 24248339; PubMed Central PMCID: PMC3856795.

32.	Christ F, Thys W, De Rijck J, Gijsbers R, Albanese A, Arosio D, et al. Transportin-SR2 imports HIV into the nucleus. Curr Biol. 2008;18(16):1192-202. Epub 2008/08/30. doi: 10.1016/j.cub.2008.07.079. PubMed PMID: 18722123.

33.	Albanese A, Arosio D, Terreni M, Cereseto A. HIV-1 pre-integration complexes selectively target decondensed chromatin in the nuclear periphery. PLoS One. 2008;3(6):e2413. Epub 2008/06/12. doi: 10.1371/journal.pone.0002413. PubMed PMID: 18545681; PubMed Central PMCID: PMC2398779.

34.	Bejarano DA, Peng K, Laketa V, Borner K, Jost KL, Lucic B, et al. HIV-1 nuclear import in macrophages is regulated by CPSF6-capsid interactions at the Nuclear Pore Complex. Elife. 2019;8. Epub 2019/01/24. doi: 10.7554/eLife.41800. PubMed PMID: 30672737.

35.	Peng K, Muranyi W, Glass B, Laketa V, Yant SR, Tsai L, et al. Quantitative microscopy of functional HIV post-entry complexes reveals association of replication with the viral capsid. Elife. 2014;3:e04114. Epub 2014/12/18. doi: 10.7554/eLife.04114. PubMed PMID: 25517934; PubMed Central PMCID: PMC4293571.

36.	Desai TM, Marin M, Sood C, Shi J, Nawaz F, Aiken C, et al. Fluorescent protein-tagged Vpr dissociates from HIV-1 core after viral fusion and rapidly enters the cell nucleus. Retrovirology. 2015;12(1):88. Epub 2015/10/30. doi: 10.1186/s12977-015-0215-z. PubMed PMID: 26511606; PubMed Central PMCID: PMC4625717.

37.	Sood C, Francis AC, Desai TM, Melikyan GB. An improved labeling strategy enables automated detection of single-virus fusion and assessment of HIV-1 protease activity in single virions. J Biol Chem. 2017. Epub 2017/10/20. doi: 10.1074/jbc.M117.818088. PubMed PMID: 29046351.

38.	McDonald D, Vodicka MA, Lucero G, Svitkina TM, Borisy GG, Emerman M, et al. Visualization of the intracellular behavior of HIV in living cells. J Cell Biol. 2002;159(3):441-52. PubMed PMID: 12417576.
